# Elastic and mechanical softening in boron-doped diamond

**DOI:** 10.1038/srep42921

**Published:** 2017-02-24

**Authors:** Xiaobing Liu, Yun-Yuan Chang, Sergey N. Tkachev, Craig R. Bina, Steven D. Jacobsen

**Affiliations:** 1 Department of Earth and Planetary Sciences, Northwestern University, Evanston, IL 60208, USA; 2 Center for Advanced Radiation Sources, University of Chicago, IL 60637, USA

## Abstract

Alternative approaches to evaluating the hardness and elastic properties of materials exhibiting physical properties comparable to pure diamond have recently become necessary. The classic linear relationship between shear modulus (*G*) and Vickers hardness (*H*_*V*_), along with more recent non-linear formulations based on Pugh’s modulus extending into the superhard region (*H*_*V*_ > 40 GPa) have guided synthesis and identification of novel superabrasives. These schemes rely on accurately quantifying *H*_*V*_ of diamond-like materials approaching or potentially exceeding the hardness of the diamond indenter, leading to debate about methodology and the very definition of hardness. Elasticity measurements on such materials are equally challenging. Here we used a high-precision, GHz-ultrasonic interferometer in conjunction with a newly developed optical contact micrometer and 3D optical microscopy of indentations to evaluate elasticity-hardness relations in the ultrahard range (*H*_*V*_ > 80 GPa) by examining single-crystal boron-doped diamond (BDD) with boron contents ranging from 50–3000 ppm. We observe a drastic elastic-mechanical softening in highly doped BDD relative to the trends observed for superhard materials, providing insight into elasticity-hardness relations for ultrahard materials.

Developments in superhard abrasives have led to synthesis of new classes of materials with physical properties reportedly comparable to diamond^1^,^2^,^3^,^4^,^5^,^6^,^7^,^8^. However, ongoing debate revolves around how to quantitatively define the hardness of such materials^9^,^10^,^11^,^12^,^13^. Hardness measurements rely on determining the projected contact area of micro- and nano-indentations using indenters made of natural diamond, but the interpretation of results from standard methods becomes problematic when cracking or plastic deformation occurs around the indentations for these brittle materials^2^,^9^,^14^. Clearly, an improved method of estimating hardness of superhard (Vicker’s hardness, *H*_*V*_ ~ 40–80 GPa) and ultrahard (*H*_*V*_ > 80 GPa) materials is highly desirable. Elastic moduli represent a fundamental physical property of materials, sensitive to structure (interatomic potentials) and composition, and in certain circumstances can be measured or calculated in a highly accurate manner^15^. While prior work has demonstrated that the intrinsic correlation between hardness and shear modulus (*G*) can correctly provide an assessment of hardness for a wide variety of crystalline materials^16^,^17^,^18^, the trend between shear moduli and hardness in the range of 80–120 GPa is not well established owing to the lack of suitable samples and methods for characterization.

Superhard and ultrahard materials are principally diamond or diamond-like compounds, including cubic boron nitride (*c*BN)^19^, BC_5_^20^, BC_2_N^19^,^21^, and BC_4_N^21^,^22^. Among these, only BC_4_N has a reported Vickers hardness exceeding 80 GPa^22^, with the exception of compact nanopolycrystalline forms (e.g. nano-cBN^1^,^4^) and diamond-cBN nanocomposites (e.g. C_x_-BN^23^,^24^). Since hardness is easily affected by crystalline defects^25^,^26^, the doping of diamond with boron leads to a system in which it is possible to study the extended trend between *H*_*V*_ and *G* in materials approaching the hardness of diamond. Therefore, heavy boron-doping in single-crystal diamond may provide insight into possible cBN-diamond solid solution (i.e. BC_x_N) elasticity-hardness trends for future materials in the ultrahard *H*_*V*_ region of 80–120 GPa. To maintain that focus, in this work we exclude discussion of Hall-Petch effects^27^ and nanoscale shear-strengthening mechanisms in *c*BN, diamond, and C_x_-BN composites^1^,^4^,^23^,^24^,^28^, but anticipate that the methods presented here can be applied to that problem in the near future.

Boron substitution in diamond modifies its physical properties, such as adding a distinctive blue color, best known in the Hope diamond^29^. Boron-doped diamond (BDD) has been widely studied on account of its semiconducting or superconducting properties at sufficiently high doping levels^30^,^31^,^32^,^33^. BDD retains the high thermal conductivity of pure diamond^31^, is inert to most chemical reagents, remains highly transparent over a wide range of the electromagnetic spectrum, and is resistant to high levels of radiation. These properties combine to make BDD an important material in potential semiconductor applications in extreme environments. Although the electrical and optical properties of BDD are well known^34^, and the compressibility of BDD is indistinguishable from that of diamond in volume-compression measurements^35^, the precise influence of moderate-to-high boron doping on the mechanical and elastic properties of single-crystal diamond has not been investigated.

Here we studied a single-crystal BDD with a compositional gradient in boron doping spanning ~50–3000 ppm (Fig. 1). The crystal BDD-D4 was synthesized at 5.5 GPa and 1380 °C with a Kovar alloy catalyst (Fe_54_Ni_29_Co_17_) and 0.5 mm cubic-octahedral diamond seed (Figure S1). Mixtures of graphite (99.99% purity) and amorphous boron (99.999% purity, 2 wt. %) were used as the carbon and dopant source. The finished crystal size was ~5 mm in longest dimension, from which thin (100) and (110) sections were obtained. The boron concentration varies continuously within each plane, permitting characterization of hardness and elasticity on the same sample to study the effect of boron incorporation on the mechanical and elastic properties of diamond. The highest boron concentration was evident from the deep blue color in the (110) section, which was used to map lattice parameters, FTIR spectra, mechanical properties, and elastic properties as a function of boron concentration, [B], estimated from lattice parameters^36^ (Figure S2), Raman and IR spectra^37^ (Figure S3). The locations of all physical properties measurements are shown in Fig. 1.

Vickers hardness measurements were conducted on the 0.185 mm thick (110) plane using standard square-pyramidal diamond indenters at a series of applied loads (see Figure S4). Diagonals of the square indenter on (110) were oriented along <100> and <110> (Figs 1, 2). We imaged the indentations using both Scanning Electron Microscopy (SEM) and 3D optical microscopy for comparison to determine the hardness values, which ranged from about 115–120 GPa in regions of low boron concentration (<300 ppm B), 105–110 GPa in regions of intermediate boron (1000–1500 ppm B), and ~100 GPa in the highest-B region (~2000–3000 ppm). In general, no indentations were observed at loads below 0.3 N, and only a crack running in the <100> direction (~3 μm in length, 0.042 μm in depth) appears on the BDD surface at 0.49 N (Fig. 2a). The length and distribution of brittle cracks increase with loading force. The hardness data at low loading force are non-linear and do not have physical meaning^9^,^10^ but are included to help evaluate the asymptotic limit of the *H*_*V*_-load curve.

SEM images show that the obtained indentions clearly differ from softer materials, which usually exhibit square indentations. Non-square indentations are observed in the SEM images (Fig. 2b–f), which have also been observed in annealed chemical vapor deposition (CVD) diamonds (*H*_*V*_  ~ 120–160 GPa)^7^. In contrast, the 3D optical microscopy images containing depth information show that the indentations exhibit high symmetry (Fig. 3), leading us to conclude that cracks can be easily mistaken for indentation edges when using only SEM images. We also imaged the diamond indenters following each set of measurements (Figure S5), and a series of standard indentations were also made in steel using the broken indenters for comparison (Figure S6), yielding no evidence for plastic deformation of the indenting tip.

Figure 3(a–e) compares the hardness data inferred from SEM versus 3D-microscopic images of the Vickers indentations at 9.8 N load. The cracking and the brittle residue around the indentation (Fig. 3b) pose a challenge to determining the true indentation edge from the two-dimensional data contained in an SEM image. We found that 3D microscopy provides the most unambiguous edge position of the indentation because the data contain depth information that allows for distinguishing the true edge from parallel cracks near the indentation (Fig. 3c and d). Figure 3e represents the load dependence of the hardness value (*H*_*V*_) in the boron-rich area (2000–3000 ppm B). We calculated the hardness values using (*d*_1_ + *d*_2_)/2 (evaluated by 3D optical microscopy) and (*d*_1_ + *d*_3_)/2 (evaluated by SEM), giving asymptotic hardness values of 102 ± 2 GPa and 88 ± 5 GPa, respectively. The hardness curve obtained by (*d*_1_ + *d*_3_)/2 behaves in an abnormal way, as it passes through a minimum and increases again with loading force above 3 N, most likely due to cracking around the indented surface. The 3D images provide a more reliable hardness value calculated by (*d*_1_ + *d*_2_)/2. Based on the above results, we find the hardness value of BDD containing ~2000–3000 ppm B reaches the asymptotic value of ~100 GPa at 9.8 N load. To reduce surface deformation effects, the maximum indenter load was used to compare the *H*_*V*_ across regions of different boron concentration.

Figure 3f shows the hardness value obtained on the (110) plane at 9.8 N in BDD with increasing boron concentration. Whereas small amounts of boron up to ~300 ppm appear to slightly increase the hardness of diamond, we find for >2000 ppm B the hardness of diamond decreases by ~15%, from ~120 GPa to ~100 GPa. The initial increase in *H*_*V*_ observed for low concentrations of boron can possibly be explained by the replacement of Ni from the catalyst with boron (Figure S3). At higher boron concentrations, the boron defects tend to aggregate^34^ and, at high enough concentration, reduce the mechanical properties of single-crystal diamond.

To investigate the elastic properties of BDD, we used two methods for comparison: Brillouin-Mandelstam spectroscopy (BMS) and GHz-ultrasonic interferometry (Figures S7–9) in conjunction with a newly developed optical contact micrometer for sample length measurements^15^. The single-crystal elastic constants (*C*_*ij*_) and moduli (*K*_S_, *G*) of BDD containing 50–300 ppm boron determined by BMS and GHz-ultrasonic interferometry are in agreement with each other and listed along with values for natural diamond in Table 1. Along with a dramatic reduction in hardness, we find increasing boron concentration reduces the elastic moduli of diamond. Compared with natural Ia diamond (*G*_0_ = 532.6 ± 0.5 GPa)^15^, the BDD-D4 measured at location BDD-1 (Fig. 1) with <300 ppm B shows *G*_0_ = 530(±2) GPa, but there is a ~3% reduction at location BDD-2 with 2000–3000 ppm B where *G*_0_ = 517(±5) GPa. Compared with natural diamond, the single-crystal moduli *C*_11_, *C*_12_ and *C*_44_ are reduced by 2.4, 2.6, and 3.3%, respectively, while both Pugh’s modulus ratio (*G*/*K*) and Pettifor’s Cauchy pressure (*C*_12_–*C*_44_)^38^ trend toward more ductile behavior^18^ with increasing boron concentration (Table 1).

There is a dramatic elastic and mechanical softening in boron-doped diamond when comparing the properties at location BDD-1 (low boron), which are essentially equivalent to diamond, with those at location BDD-2, where boron concentrations are in excess of 2000 ppm. The precipitous drop in *H*_*V*_ in elastic-mechanical properties is most likely associated with isolated boron atoms replacing C in the diamond lattice at low concentrations (<300 ppm) and with cluster defects at higher concentrations, of which B_4_C, B_13_C_2_, and B_50_C_2_ have been proposed^34^. To illustrate the effect of boron substitution on hardness-elasticity relationships in comparison to other B-C-N compounds, Fig. 4 examines the relationships between *H*_*V*_ and elastic moduli in linear^16^ and non-linear^17^ formulations.

Figure 4a shows the highest reported *H*_*V*_ for each compound, focused on experimental data, with a vertical line representing the range in reported values (see Table S3 for references in Fig. 4). The classical linear relationship^6^ that holds well for materials with *H*_*V*_ < 40 GPa is represented by the relation, *H*_*V*_ = 0.151 *G*, derived assuming a purely elastic response to the square-pyramidal indenter^17^. Superhard materials (*H*_*V*_ > 40 GPa) generally fall above this line, but are highly scattered. Beginning with *c*BN and those with *H*_*V*_ > 60 GPa, we observe an exponential rise in *H*_*V*_ with *G* according *H*_*V*_ = 14*e*^(0.0038*G*)^, which has no theoretical foundation but is meant to simply serve as a guide for the eye in Fig. 4a. At *G* = 532 GPa, representing diamond, its value at ~105 GPa is still below the highest reported *H*_*V*_ for diamond (~120 GPa)^14^ and BDD-1 taken on the (110) plane, suggesting an abrupt jump on approach to pure diamond. We note that the current analysis does not include nano-scale strengthening effects, such as observed in nano-twinned materials^1^,^2^, which are expected to exhibit a different trend once elastic moduli for those materials can be determined. We also note that focusing on the average *H*_*V*_ value, rather than the highest reported value, would produce a different trend through the B-C-N compounds more closely resembling the linear value (*H*_*V*_ = 0.151 G)^17^, in which case there would be an even larger jump in *H*_*V*_ in going from B-C-N solid solution up to BDD and pure diamond.

A very successful formulation by Chen^17^ spanning softer and hard materials is based on Pugh’s modulus, *k* = *G*/*K*, where *K* is the bulk modulus. In particular, the relation *H*_*V*_ = 2(*k*^2^*G*)^0.585^–3 was derived and fitted to a larger dataset^17^ (not shown here) but added to Fig. 4b for comparison with our dataset (Table S3), including BDD. All materials with highest *H*_*V*_ > 40 GPa, with the exception of B_4_C and *c*BN, plot above Chen’s formulation. As a guide to the eye, we fitted a linear trend through B-C-N compounds with *H*_*V*_ > 60 GPa, finding *H*_*V*_ = 27.2 + 0.097 *G* provides an excellent fit to the B-C-N series including BBD-2. A large gap in hardness-elasticity still persists between BC_4_N and BBD, providing a goal for pure compounds in the series.

In conclusion, we have shown it is meaningful to obtain conventional Vickers hardness values in superhard materials by examining indentations with 3D optical microscopy. High-precision ultrasonic methods such as GHz-ultrasonic interferometry can constrain their elastic properties to within better than ± 1%, even in opaque materials where light-scattering techniques are problematic. We have found that highly boron-doped, single-crystal diamond displays dramatically reduced elastic and mechanical properties when compared with pure diamond but serves to fill in a critical gap in elasticity-hardness relations between BC_x_N and pure diamond. We find a highly non-linear relationship between *H*_*V*_ and *G* for materials with *H*_*V*_ > 60 GPa, best fitted empirically with an exponential function, although there is still a drastic jump to the highest *H*_*V*_ observed in diamond. In the Chen formulation^17^, B-C-N compounds are well modeled, but an empirical linear relation between BC_5_ and BBD provides a rather tight fit to the materials that undergo plastic deformation and brittle cracking when indented by square-pyramidal diamond. These relationships serve as a guide for future design and synthesis of novel abrasives and semi-conducting compounds for extreme environments in the ultrahard class of materials.

## Methods

### Sample preparation

**Figure 1:**
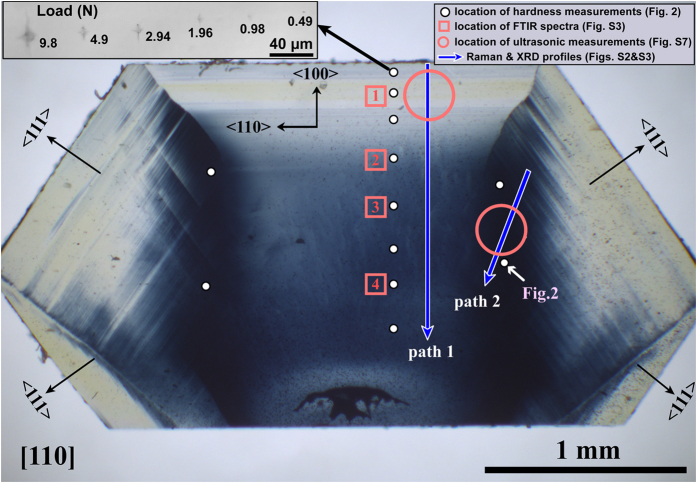
Section polished parallel to (110) of boron-doped diamond sample D4 (BDD-D4) measuring 0.185 mm thick. Vickers hardness measurements were made at every location shown by the white dots on the (110) plane with diagonals of the square pyramidal indenter parallel to <100> and <110>. FTIR spectra were obtained at locations 1–4 shown by the red boxes, and GHz-ultrasonic measurements of elastic properties were made at locations labeled BDD-1 and BDD-2, bounded by the red circles. A schematic illustration of these measurements is shown in Figure S4. X-ray diffraction and Raman measurements shown in Figures S2 and S3 were taken along paths represented by the blue arrows.

The BDD crystals D2 and D4 were synthesized at 5.5 GPa and at ~1300 and ~1380 °C, respectively, by the temperature gradient growth method in a China-type cubic high-pressure apparatus at State Key Laboratory of Superhard Materials of Jilin University. A Kovar alloy (Fe_54_Ni_29_Co_17_) was used as the catalyst and cubo-octahedral diamond (500 *μm*) as the seed. Mixtures of graphite (99.99% purity) and amorphous boron (99.999% purity) were used as carbon and dopant source (~0.5 wt.% boron for D2 and ~2 wt.% boron for D4). From sample D4, (110) and (100) sections measuring 0.185 mm and 0.31 mm thickness, respectively, were laser cut and polished by Almax-easyLab (Figure S1). The boron concentration gradient was estimated from Raman spectrum (Figure S3) and lattice parameters (Figure S2) measured by synchrotron X-ray diffraction at sector 16-BM-D of the Advanced Photon Source (APS), Argonne National Laboratory. Synchrotron-IR measurements were conducted at beamline U2A of the National Synchrotron Light Source (NSLS), Brookhaven National Laboratory. IR spectra (Figure S3a) were collected from 400 to 5000 cm^−1^ with a spectral resolution of 1 cm^−1^ in transmittance mode with a ~15 × 15 μm aperture in selected regions shown in Fig. 1.

### Hardness measurement

**Figure 2:**
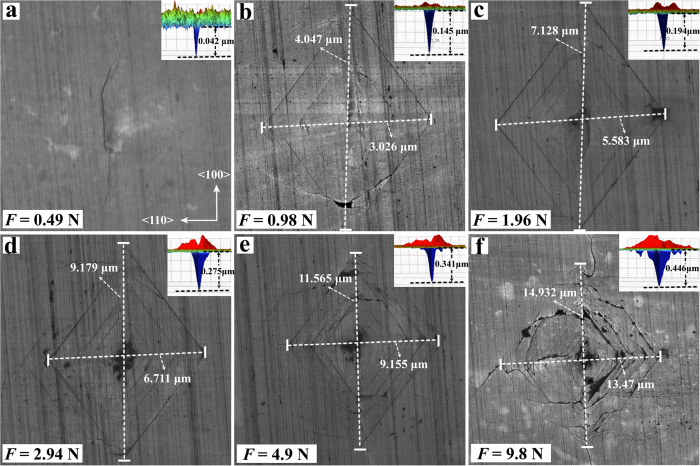
**(a–f)** SEM and 3D optical microscopic images (inset) of indentations produced on the (110) plane of BDD-D4 shown in Fig. 1 at a location of high boron concentration shown by the white arrow in Fig. 1. The load used for each indentation in (**a–f)** is shown in the lower-left corner of each panel.

Vickers hardness was performed using a micro-hardness tester (Duramin-20, Struers) under loads from 0.1 to 10 N (Fig. 2). Hardened steel was used as a standard before and after diamond measurements (Figure S6). Indentations were studied by SEM and with a high-resolution 3D microscope (Bruker, ContourGT Optical Profiler).

### Elastic property measurement

Brillouin-Mandelstam spectroscopy was performed at beamline 13-ID-B (GSECARS) of the APS. GHz-ultrasonic interferometry, in conjunction with the newly developed optical contact micrometer for high-precision length measurements, was conducted at Northwestern University.

## Additional Information

**How to cite this article**: Liu, X. *et al*. Elastic and mechanical softening in boron-doped diamond. *Sci. Rep.* **7**, 42921; doi: 10.1038/srep42921 (2017).

**Publisher's note:** Springer Nature remains neutral with regard to jurisdictional claims in published maps and institutional affiliations.

## Supplementary Material

Supplementary Information

## Figures and Tables

**Figure 3:**
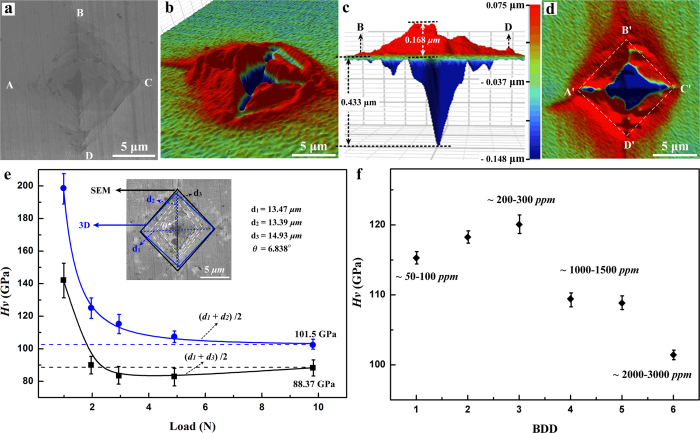
Hardness measurements of boron-doped diamond crystal BDD-D4 were analyzed by **(a)** SEM and **(b–d)** 3D optical microscopy for comparison. Because the SEM image is two dimensional, it can be difficult to distinguish cracking from the true edge of the indentation. Using 3D microscopy, depth information is used to determine the edge position. **(e)** *H*_*V*_ of BDD in a region of high boron concentration (2000–3000 ppm) as a function of applied load using edge lengths determined by SEM (black) and by 3D optical microscopy (blue). **(f)** Dependence of Vickers hardness on boron content.

**Figure 4:**
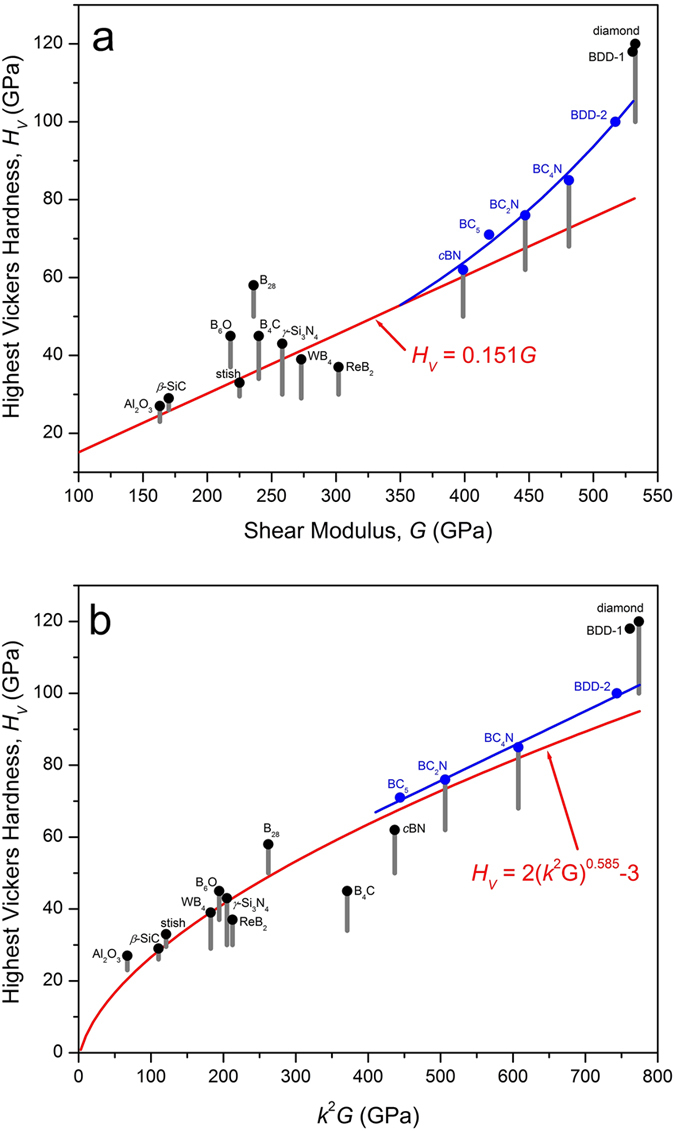
(**a**) Variation of hardness with shear modulus for a set of mostly experimental data presented in Table S3. An empirical non-linear correlation for materials with *H*_*V*_ > 60 GPa is shown as a guide to the eye with *H*_*V*_ = 14*e*(0.0038 *G*). (**b**) Chen’s formulation^7^ compared with a linear fit to B-C-N compounds with *H*_*V*_ > 60 GPa (Table S3). Nano-scale strengthening effects, such as those observed in nanotwinned (NT) materials such as NT-cBN^1^ and NT-diamond^2^, are not plotted because their elastic moduli have not been determined.

**Table 1:** Elastic^a^ and mechanical^b^ properties of diamond and boron-doped diamond (BDD).

	Single-crystal Ia diamond^39^	Single-crystal Ia diamond^15^	BDD (50–300 ppm)^c^	BDD-1 (50–300 ppm)^d^	BDD-2 (2000–3000 ppm)^d^
Method	MHz-ultrasonic	GHz-ultrasonic	Brillouin	GHz-ultrasonic	GHz-ultrasonic
*C*_11_ (GPa)	1076 (±5)	1074.8 (±0.4)	1082 (±6.8)	1082.0 (±4.8)	1049.5 (±9.6)
*C*_12_ (GPa)	125 (±6)	125.3 (±1.0)	125 (±12.8)	123.2 (±4.8)	122.0 (±9.6)
*C*_44_ (GPa)	576 (±2)	575.2 (±0.3)	571 (±5.416)	567.36 (±0.2)	556.1 (±2.0)
*ρ* (kg/m^3^)	3512 (±1)	3512 (±1)	3513 (±1)	3513 (±1)	3503 (±1)
*K*_S_ (GPa)	442.0 (±5.7)	441.8 (±0.8)	444 (±8.8)	442.8 (±4.8)	431.2 (±9.6)
*G* (GPa)	533.4 (±3.6)	532.6 (±0.5)	532 (±3.1)	530.5 (±2.2)	517.1 (±5.4)
*E* (GPa)	1141 (±10)	1140 (±1)	1143 (±29)	1137 (±7)	1108 (±15)
*H*_*V*_ (GPa)^b^				118 (±3)	100 (±2)

^a^Elastic properties: *C*_ij_, single-crystal elastic constants; *K*_0S_, adiabatic bulk modulus; *G*_0_, shear modulus; *E*, Young’s modulus, calculated from *C*_ij_ according to the Hill average of the Voigt-Reuss bounds.

^b^Vickers hardness, *H*_*V*_.

^c^This study, measured in the (110) section of crystal BDD-D2 in an area of low boron concentration (Figures S7 and S9).

^d^This study, measured in the (110) section of sample BDD-D4 at locations BDD-1 and BDD-2, shown in Fig. 1.
