# Supplementary material for: Elastic and mechanical softening in boron-doped diamond
Source: Sci Rep. 2017 Feb 24;7:42921. doi: 10.1038/srep42921 (PMC5324052; doi:10.1038/srep42921)
Supplement: Supplementary Information [file srep42921-s1.pdf]

Supplementary Information

Elastic and mechanical softening in boron-doped diamond

Xiaobing Liu[1*], Yun-Yuan Chang[1], Sergey N. Tkachev[2], Craig R. Bina[1] and Steven D. Jacobsen[1]

[1]*Department of Earth and Planetary Sciences, Northwestern University, Evanston, IL 60208, USA*

[2]*Center for Advanced Radiation Sources, University of Chicago, IL 60637, USA*

* ***Email:*** *xiaobing@earth.northwestern.edu*

[†] *Current address: Institute of Earth Sciences, Academia Sinica, Nangang, Taipei 11529, Taiwan*

This Supplement includes:

Supplementary Figures S1-S9

Supplementary Tables S1-S3

Supplementary References

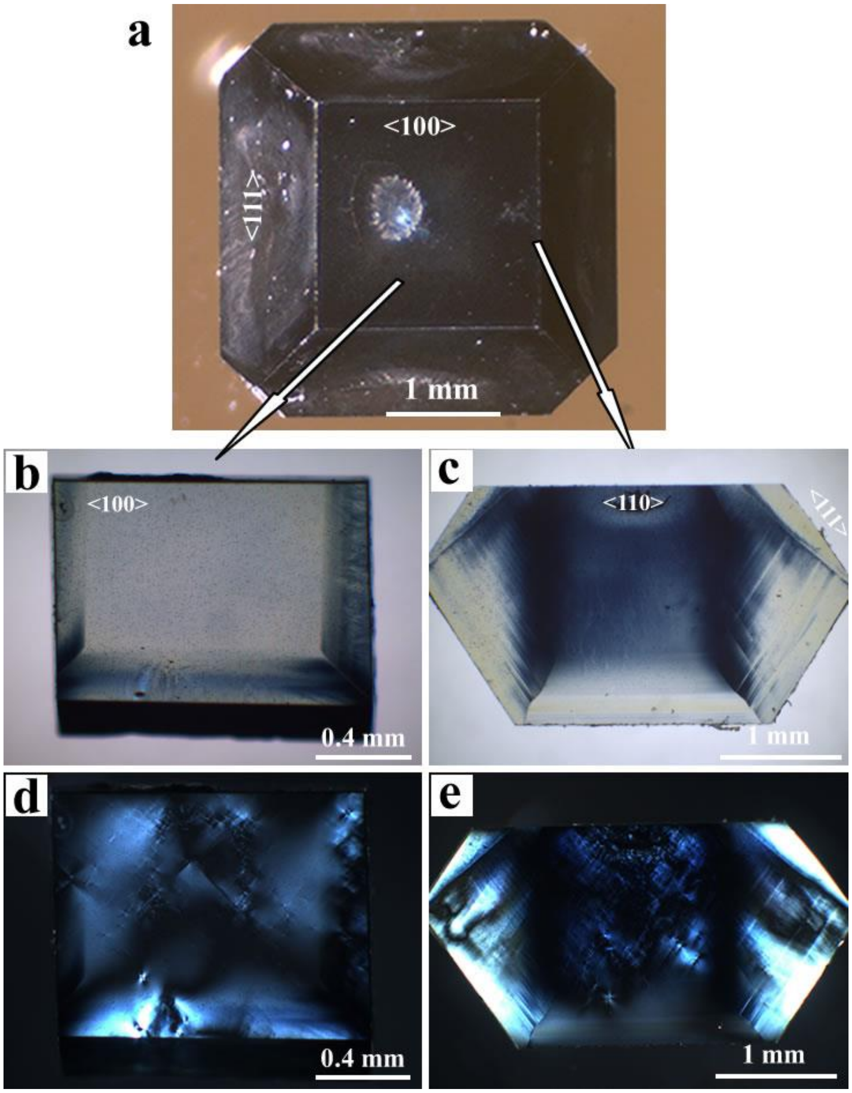

Figure S1. (a) Photomicrograph of the HPHT boron-doped diamond (BDD) sample D4, synthesized using the temperature-gradient growth method at 5.5 GPa and 1380 °C in a large-volume, cubic press at State Key Laboratory of Superhard Materials, Jilin University, China. Sections (100) and (110) were laser cut and polished from the original crystal by Almax-easyLab, shown in **b-d**. Transmitted light photomicrographs of the (100) and (110) sections are shown in panels **b** and **c**, respectively. Cross-polarized light photomicrographs of the (100) and (110) sections are shown in **d** and **e**, respectively. The (100) section is ~0.31 mm thick, and the (110) section is ~0.18 mm thick.

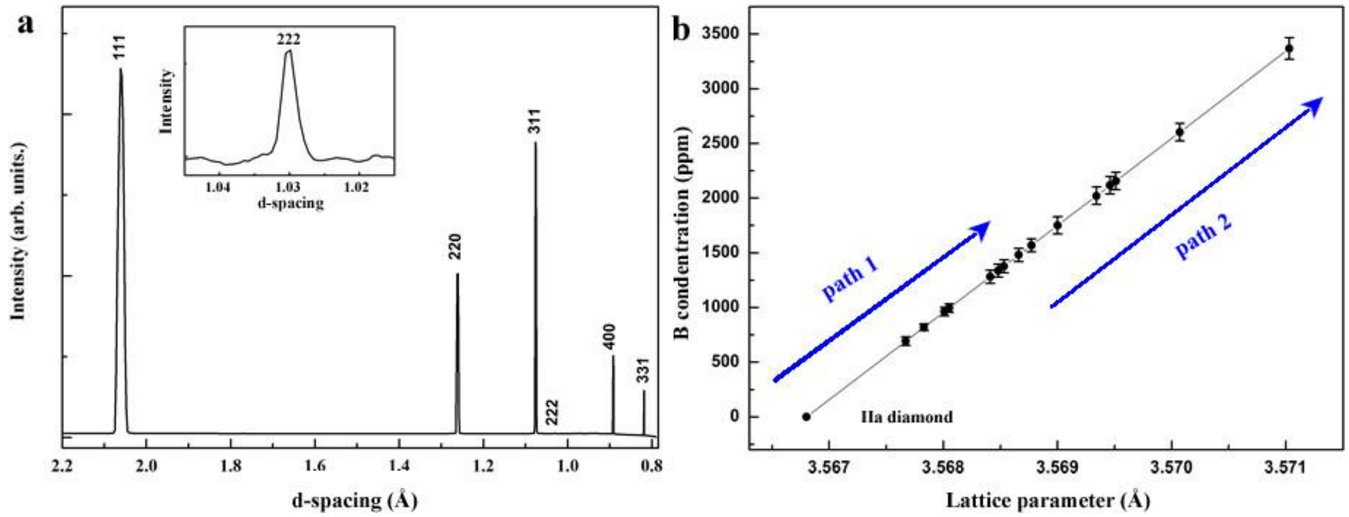

Figure S2. (a) X-ray diffraction pattern of the BDD-D4 taken in a region of high boron concentration [B], integrated from a single-crystal CCD image. **(b)** Variation of the lattice parameter measured from low to high boron concentration in the (110) slice along paths 1 and 2, shown in Figure 1. Boron concentrations were estimated using the calibration of Brunet *et al*. (1998).

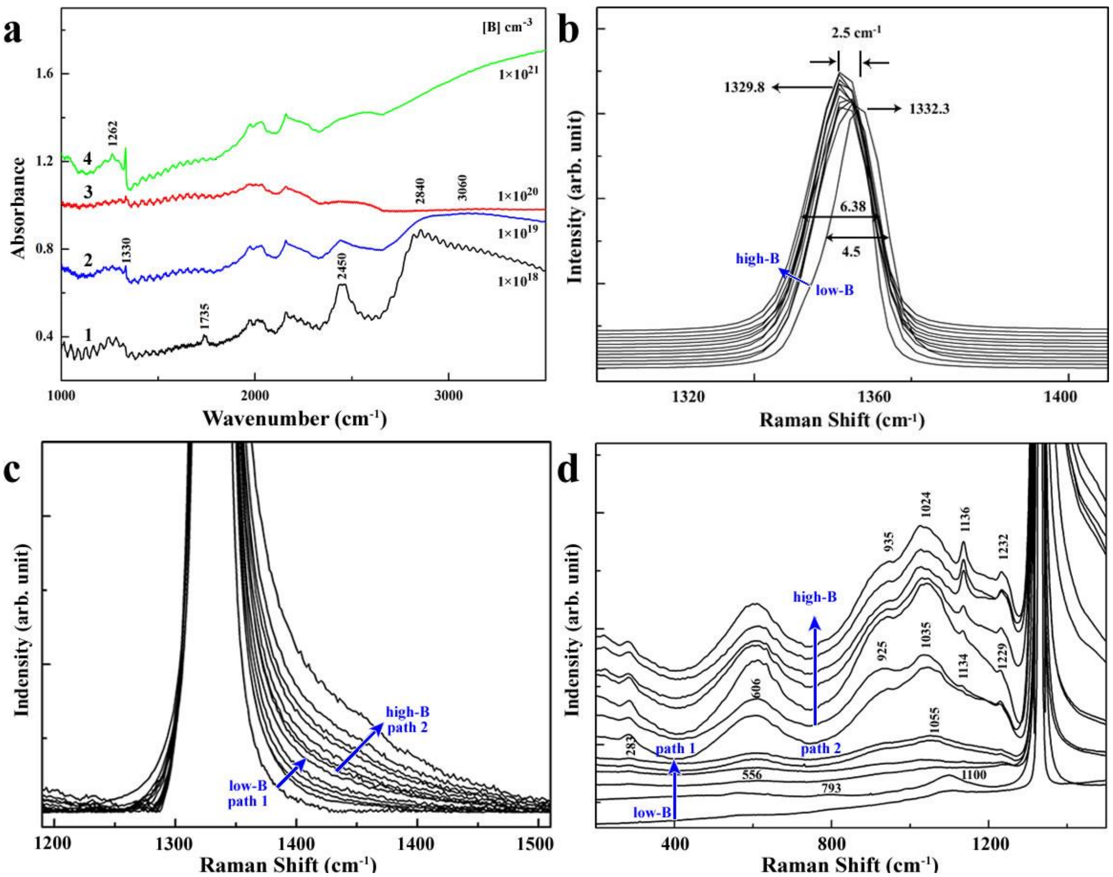

Figure S3. **(a)** FTIR spectra of the BDD-D4 sample ((110) section, thickness 0.185 mm) taken at locations labeled 1-4 in Figure 1. Spectra 2-4 are offset for clarity. The peaks labeled 2450, 2840, and the broad shoulder at ~3060 cm^{-1} are assigned to transitions between bound states of boron acceptors, which broaden with increasing boron concentration, [B] (Ager *et al*. 1995). **(b)** First-order longitudinal phonons in BDD, measured by Raman spectroscopy along paths 1 and 2 shown in Figure 1 from low [B] (1332.2 cm^{-1}) to high [B] (1329.8 cm^{-1}), are accompanied by broadening of the FWHM from ~4.5 to 6.4 cm^{-1} due to tensile stress in the diamond lattice with increasing [B]. **(c)** Line broadening is observed on the high-wavenumber side of the ~1332 cm^{-1} vibration with increasing [B], attributed to a Fano-type interference (Ager *et al*. 1995) between the discrete zone-center phonon and the continuum of electronic states induced by the presence of the dopant in the boron-rich region. **(d)** Raman spectra of BDD along the two paths shown in Figure 1: at [B] below ~10^{19} cm^{-3}, the 1100 cm^{-1} peak is produced by symmetric Ni-O vibrations introduced by the Kovar alloy catalyst and disappears with increasing of B in diamond. This indicates that the boron dopants can take the position of substituted nickel in diamond, leading to an improvement of H_V in the low-B region (Figure 3f). At higher [B] contents (path 2) a series of new peaks appear in the spectra, notably the sharp peaks labeled 1134-1136 cm^{-1} and 1229-1232 cm^{-1}, and several broader peaks at around 283, 606, 925, and 1071 cm^{-1}, all of which grow in intensity with increasing [B]. All of the spectroscopic data illustrate that boron concentration changes continuously in the same crystallographic orientation from tens or hundreds of ppm (~10^{18} cm^{-3}) in the low-boron regions to thousands of ppm (~10^{21} cm^{-3}) in the high-boron regions.

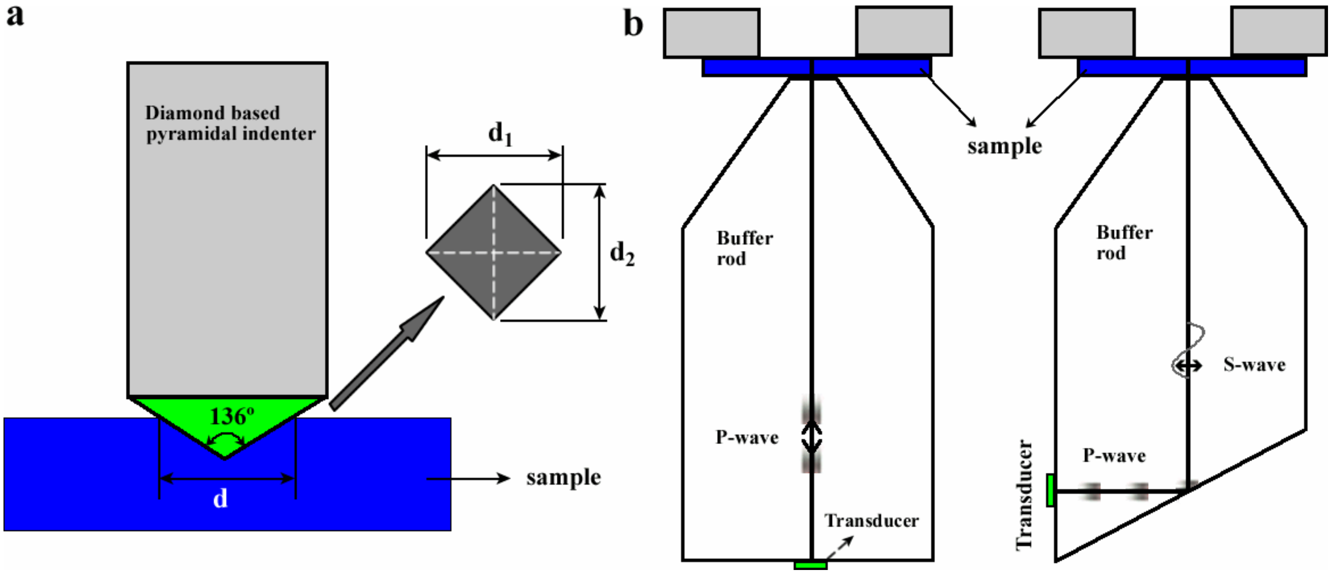

Figure S4. Schematic diagrams of the hardness measurement **(a)** and the GHz-ultrasonic interferometry experiment **(b)** with P-wave (left) and S-wave (right).

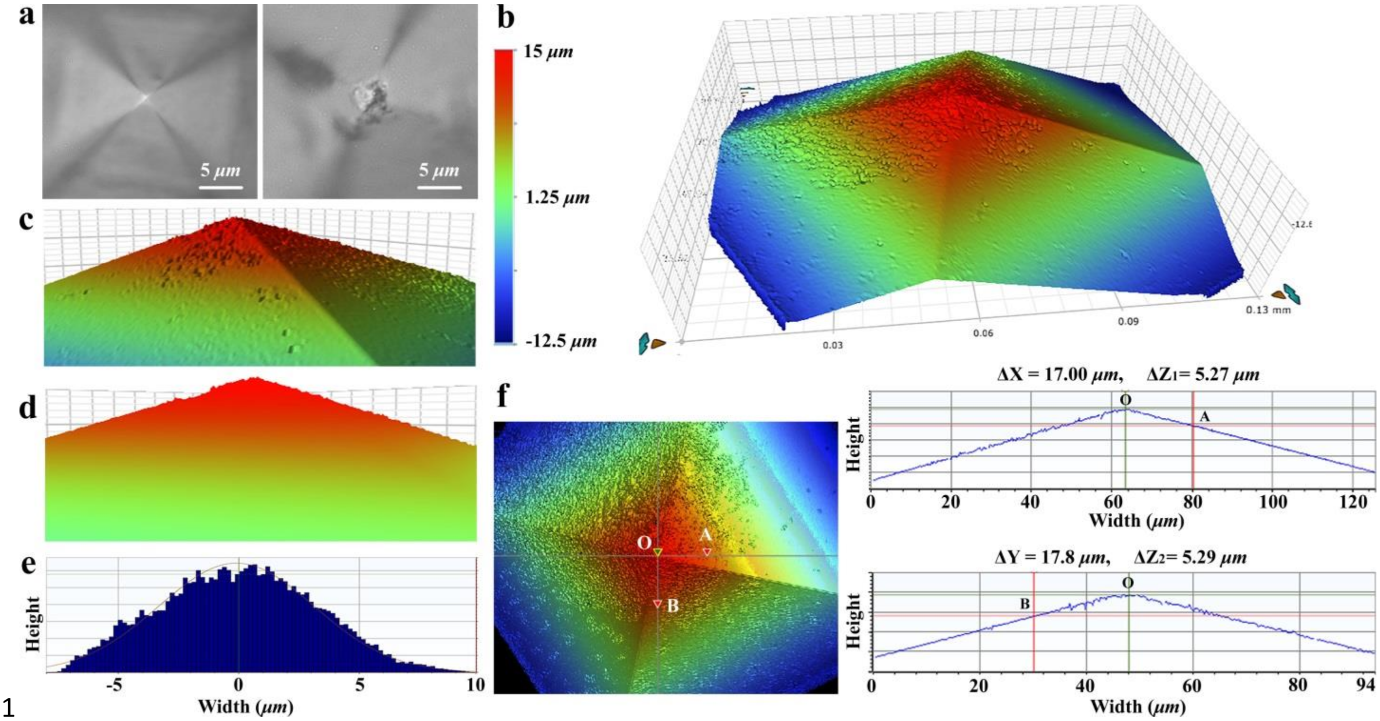

Figure S5. Optical photomicrographs **(a)** of a diamond indenter tip before and after damage from loading at 9.8 N on BDD-D4. Analysis of the broken tip was conducted using 3D optical microscopy **(b-f)**, showing brittle cracking without plastic deformation while it remains highly symmetric.

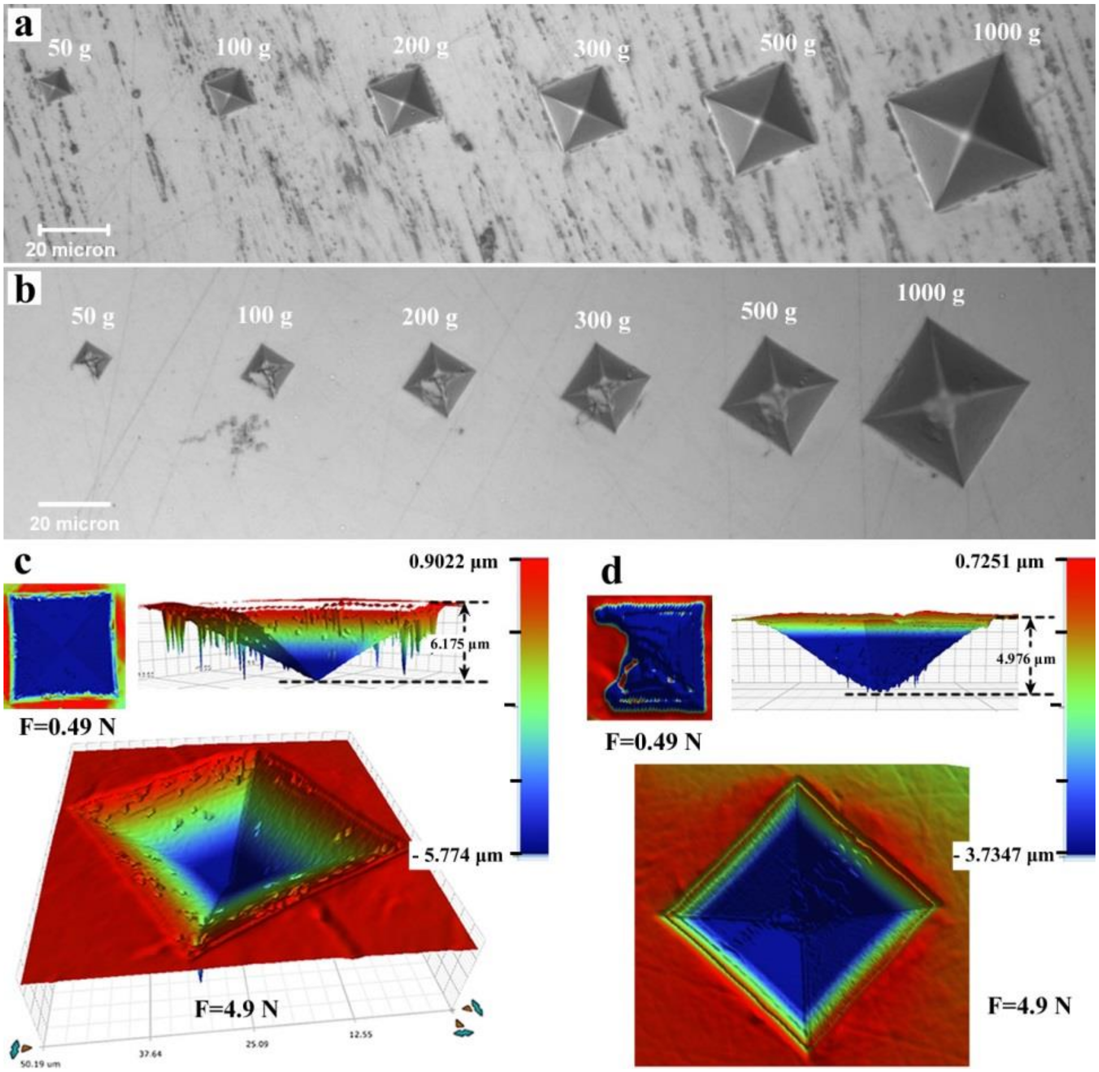

Figure S6. Comparison of indentations formed following a series of applied loads on a steel standard using **(a)** new and **(b)** damaged diamond indenters. The 3D structure of the indentations produced at 0.49 N and 4.9 N is shown in **c** and **d**. These calibration results on indenter tips and indentations further ensures that there is no plastic deformation of the indenter tip following hardness testing of boron-doped diamond.

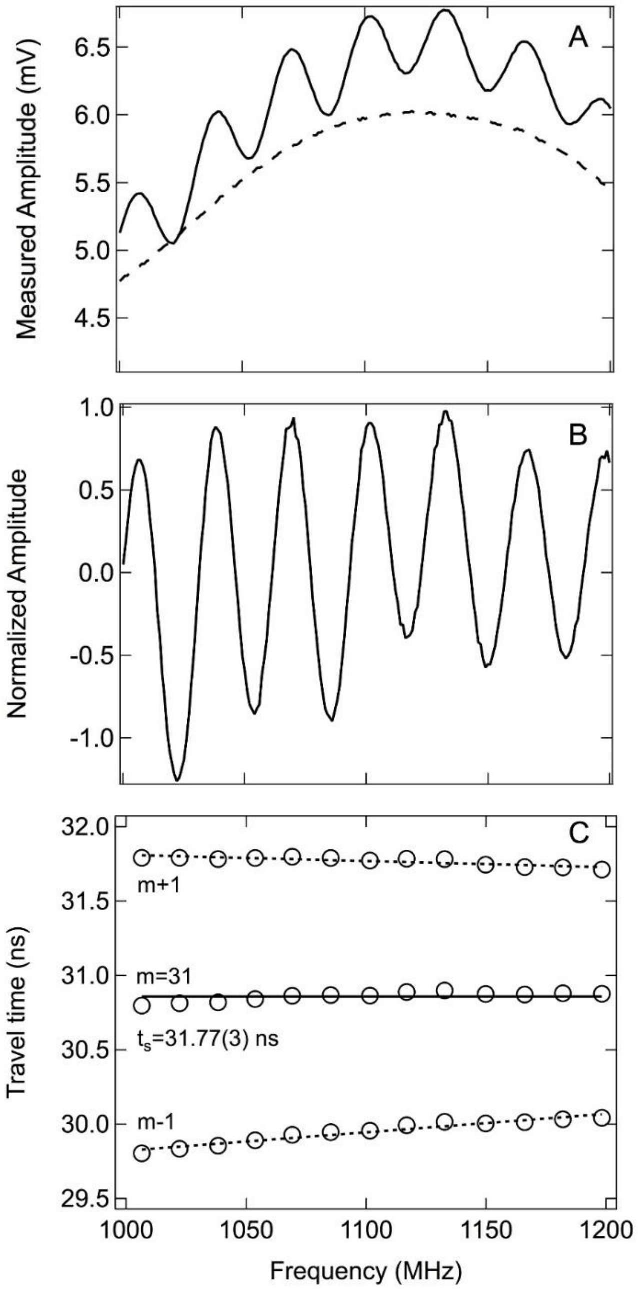

Figure S7. Example GHz-ultrasonic interferometry S-wave dataset measured at location BDD-1 shown in Figure 1: **(a)** solid line shows the interfered amplitude of tone bursts from the near- and far-side of the polished sample from 1-1.2 GHz. Dashed line shows the un-interfered sample echo from the near-side of the sample, which is used to demodulate the interfered spectrum, shown in panel **b**. Round-trip travel times as a function of frequency for each pair of maxima and minima (Δf) using m = 31, the correct integer number of acoustic wavelengths in the first maximum, are shown in **c**. All ultrasonic travel-time data are given in Tables S1 and S2.

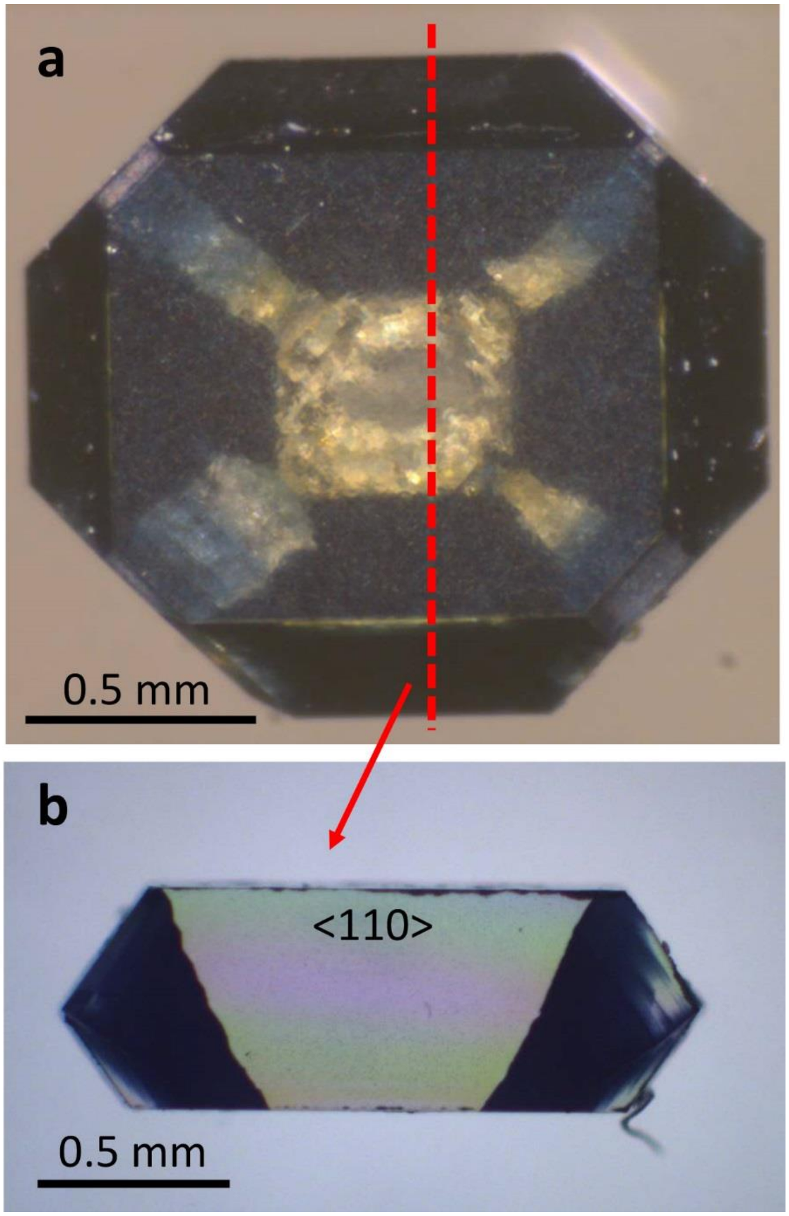

Figure S8. (a) Photomicrograph of the HPHT boron-doped diamond (BDD) sample D2, synthesized using the temperature-gradient growth method at 5.5 GPa and ~1300 °C in a large-volume, cubic press at State Key Laboratory of Superhard Materials, Jilin University, China. **(b)** a section (110) was laser cut and polished from the original crystal by Almax-easyLab, represented by the red dashed line in a, and shown in transmitted light in b. The Brillouin scattering measurement was made in a low-boron region of this sample, containing < 300 ppm boron.

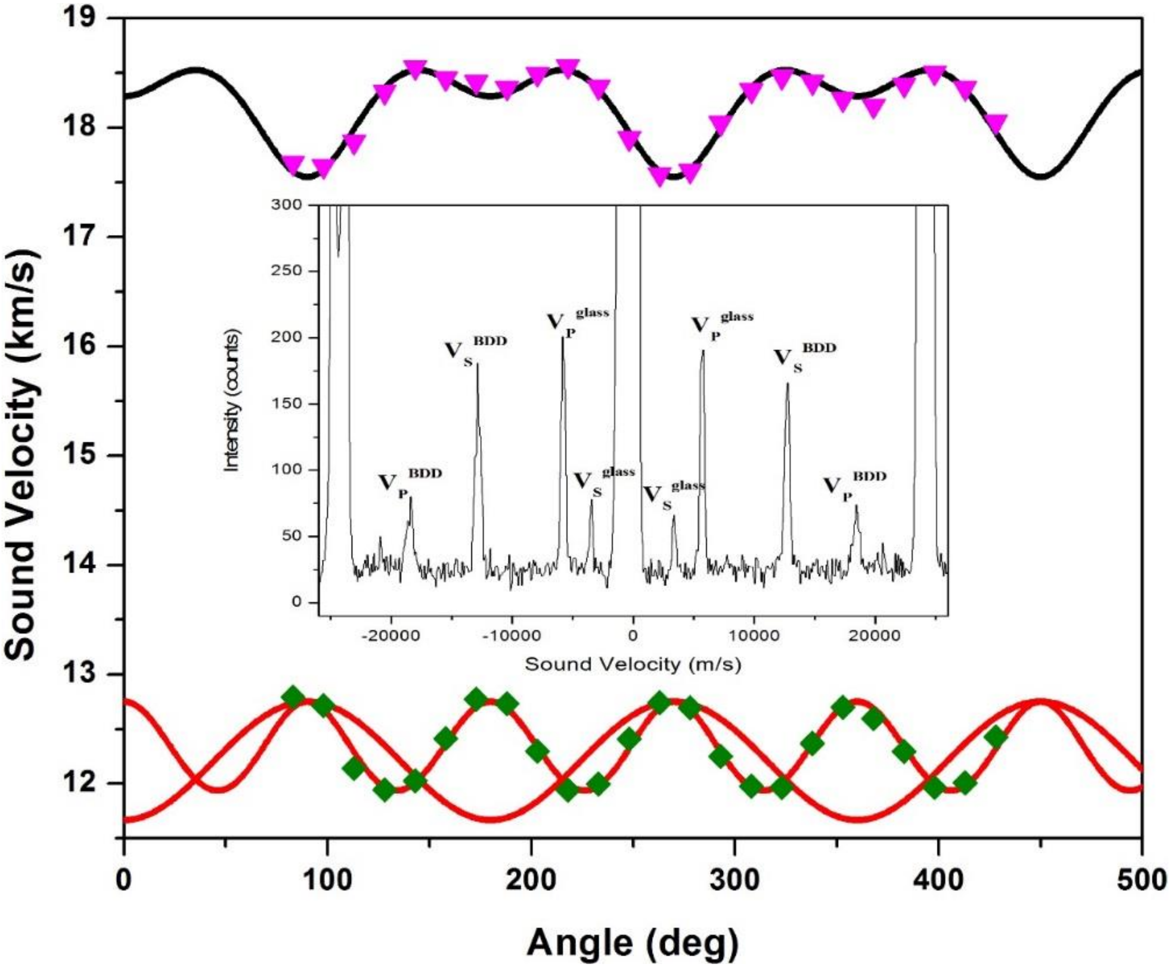

Figure S9. Longitudinal (triangles) and transverse (diamonds) acoustic velocities of BDD sample D2, measured by Brillouin-Mandelstam spectroscopy in a low-boron region of the (110) section shown in Figure S7 (< 300 ppm boron). Fitted anisotropy curves (solid lines) were calculated on the basis of the best-fit model for the elastic constants. Errors of the measurements are not larger than the size of the symbols. A typical Brillouin spectrum of BDD-D2 contained between two glass slides is shown in inset.

Table S1. GHz-ultrasonic travel time and velocity data for BDD (sample D4) at location BDD-1 shown in Figure 1 (low boron concentration, 50-300 ppm).

	Location BDD-1 (50-300 ppm)		
	Travel time (ns)	Thickness (mm)[a]	Velocity (m/s)
P-wave measurement: $V_p{}^{[110]}{}_{pol[110]}$			
Measurement 1	20.34(±0.02)	0.18550(±0.00005)	18241.4(± 19.3)
Measurement 2	20.32(±0.01)	0.18550(±0.00005)	18260.5(±10.2)
		Average $V_p{}^{[110]}{}_{pol[001]}$	18251.0(±14.8)
S-wave measurement: $V_S{}^{[110]}{}_{pol[101]}$			
Measurement 1	31.65(±0.02)	0.18550(±0.00005)	11721.2(± 9.6)
Measurement 2	31.85(±0.03)	0.18550(±0.00005)	11647.8(±13.1)
Measurement 3	31.77(±0.03)	0.18550(±0.00005)	11676.1(±12.4)
		Average $V_S{}^{[110]}{}_{pol[101]}$	11681.7(±11.7)
S-wave measurement: $V_S{}^{[110]}{}_{pol[001]}$			
Measurement 1	29.18(±0.02)	0.18550(±0.00005)	12712.1(± 7.8)
Measurement 2	29.19(±0.01)	0.18550(±0.00005)	12710.3(± 6.9)
Measurement 3	29.18(±0.01)	0.18550(±0.00005)	12712.1(± 5.5)
Measurement 4	29.19(±0.03)	0.18550(±0.00005)	12707.9(±12.1)
		Average $V_S{}^{[110]}{}_{pol[001]}$	12710.6(±8.1)

[a]Thickness measurements were made using the optical contact micrometer described in Chang *et al.* (2014) at the location of BDD-1 shown in Figure 1.

Table S2. GHz-ultrasonic travel time and velocity data for BDD (sample D4) at location BDD-2 shown in Figure 1 (high boron concentration, 2000-3000 ppm).

	Location BDD-2 (2000-3000 ppm B)		
	Travel time (ns)	Thickness (mm)[a]	Velocity (m/s)
P-wave measurement: $V_p{}^{[110]}{}_{pol[110]}$			
Measurement 1	20.66(±0.03)	0.18600(±0.00005)	18009.3(± 30.7)
Measurement 2	20.55(±0.03)	0.18600(±0.00005)	18102.7(±28.0)
Measurement 3	20.61(±0.06)	0.18600(±0.00005)	18052.0(±49.5)
		Average $V_p{}^{[110]}{}_{pol[001]}$	18054.7(±36.1)
S-wave measurement: $V_S{}^{[110]}{}_{pol[101]}$			
Measurement 1	32.41(±0.03)	0.18600(±0.00005)	11479.4(± 12.7)
Measurement 2	32.18(±0.03)	0.18600(±0.00005)	11559.4(±12.9)
Measurement 3	32.41(±0.03)	0.18600(±0.00005)	11479.4(±12.7)
		Average $V_S{}^{[110]}{}_{pol[101]}$	11506.1(±12.8)
S-wave measurement: $V_S{}^{[110]}{}_{pol[001]}$			
Measurement 1	29.47(±0.01)	0.18600(±0.00005)	12624.7(± 6.4)
Measurement 2	29.60(±0.02)	0.18600(±0.00005)	12569.6(± 8.8)
Measurement 3	29.54(±0.03)	0.18600(±0.00005)	12594.7(± 13.8)
Measurement 4	29.50(±0.02)	0.18600(±0.00005)	12610.7(±7.6)
		Average $V_S{}^{[110]}{}_{pol[001]}$	12599.9(±9.2)

[a]Thickness measurements were made using the optical contact micrometer described in Chang *et al.* (2014) at the location of BDD-2 shown in Figure 1.

Table S3. Shear modulus (*G*), bulk modulus (*K*), and Vickers hardness (H_V) in units of GPa, plotted in Figure 4 with references [ref][a] given in brackets.

**Phase**	**G_0 [ref]**	**K_0 [ref]**	**H_V [ref]**
diamond	532.6 [1] (Ia)	441.8 [1] (Ia)	90-120 [2] (IIa)
BDD-1<br>50-300 ppm B	530.5 [3]	442.8 [3]	118 [3]
BDD-2<br>2000-3000 ppm B	517.1 [3]	431.2 [3]	100 [3]
BC_4N	481 [4]	428 [4]	68 [5] – 85 [6]
BC_2N	447 [7]	420 [4]	62 [5] – 76 [7]
BC_5	419 [8]	407 [8]	71 [9]
*c*BN	398.8 [10]	381.1 [10]	50 [11] – 62 [6]
γ-B_{28}	236 [12]	224 [12]	50 [13] – 58 [14]
B_6O	218 [15]	231 [15]	37 [16] – 45 [17]
γ-Si_3N_4	258.3 [18]	290 [19]	30 – 43 [19] 43 [20]
stishovite-SiO_2	225 [21]	307 [21]	29.5 [22] – 33 [23]
ReB_2	302 [24]	360 [24]	30 [25] – 37 [24]
β-SiC	170 [26]	211 [26]	26-28 [27] 29 [28]
B_4C	240 [29]	193 [29]	34 [16] – 45 [30]
WB_4	273 [31]	334 [31]	29 [32] – 39 [31]
Al_2O_3	163.1 [33]	253.7 [33]	23 [34] – 27 [35]

References: [1] Chang *et al.* 2014; [2] Yan *et al.* 2004; [3] This study; [4] Chen *et al.* 2008; [5] Zhao *et al.* 2002; [6] Tang *et al*. 2012; [7] Solozhenko *et al.* 2001; [8] Zhao and Wang 2011; [9] Solozhenko *et al.* 2009; [10] Zhang *et al.* 2011a; [11] Sumiya *et al.* 2014; [12] Jiang *et al.* 2009; [13] Solozhenko *et al.* 2008; [14] Zarechnaya *et al.* 2009; [15] Zhang *et al.* 2011b; [16] Rizzo *et al.* 1967; [17] He *et al.* 2002; [18] Soignard *et al.* 2001; [19] Zerr *et al.* 2002; [20] Tanaka *et al.* 2002; [21] Yoneda *et al.* 2012; [22] Nishiyama *et al.* 2014; [23] Léger *et al.* 1996; [24] Chung *et al.* 2008; [25] Dubrovinskaia *et al.* 2007; [26] Li and Bradt 1987; [27] Sung *et al.* 1996; [28] Dubrovinsky *et al.* 2001; [29] Dodd *et al.* 2002; [30] Mukhanov *et al.* 2010; [31] Li *et al.* 2016; [32] Cheng *et al.* 2014; [33] Goto *et al.* 1989; [34] Evans and Charles 1976; [35] Krell 1980.

Supplemental references

Ager III, J.W., Walukiewicz, W., McCluskey, M., Plano, M.A., & Landstrass, M. Fano interference of the Raman phonon in heavily boron-doped diamond films grown by chemical vapor deposition, *Appl. Phys. Lett.* **66**, 616-618 (1995).

Brunet, F.; Germi, P.; Pernet. The effect of boron doping on the lattice parameter of homoepitaxial diamond films, *Diam. Relat. Mater.* **7**, 869, (1998).

Chang, Y.-Y., Jacobsen, S. D., Kimura, M., Irifune, T. & Ohno, I. Elastic properties of transparent nano-polycrystalline diamond measured by GHz-ultrasonic interferometry and resonant sphere methods. *Phys. Earth Planet. Inter.* **228**, 47-55, (2014).

Chen S., Gong X.G., & Wei S.H. Crystal structures and mechanical properties of superhard BC_2N and BC_4N alloys: First-principles calculations. *Phys. Rev. B* **77**, 014113, (2008).

Cheng, X.Y., Chen, X.Q., Li, D.Z. & Li, Y.Y, Computational materials discovery: The case of the W-B system, *Acta Crystallogr. Sect. C*, **70**, 85-103, (2014).

Chung, H.Y., *et al.*, Correlation between hardness and elastic moduli of the ultraincompressible transition metal diborides RuB_2, OsB_2, and ReB_2, *Appl. Phys. Lett.* **92**, 261904 (2008).

Dodd, S.P., Saunders, G.A. & James, B., Temperature and pressure dependences of the elastic properties of ceramic boron carbide (B_4C), *J. Mater. Sci.*, **37**, 2731-2736, 2002.

Dubrovinsky, N.A. & Dubrovinsky, L.S., High-pressure silica polymorphs as hardest known oxides, *Mater. Chem. Phys.*, **68**, 77-79, (2001).

Dubrovinskaia, N., Dubrovinsky, L. & Solozhenko, V.L., Comment on "Synthesis of Ultra-Incompressible Superhard Rhenium Diboride at Ambient Pressure", *Science*, **318**, 1550, (2007).

Evans, A.G. & Charles, E.A., Fracture toughness determinations by indentation, *J. Am. Ceram. Soc.*, **59**, 371-372, (1976).

Goto, T., Anderson, O.L., Ohno, I., & Yamamoto, S., Elastic constants of corundum up to 1825 K, *J. Geophys. Res.: Solid Earth*, **94**, 7588-7602, (1989).

He, D.W., *et al.*, Boron suboxide: As hard as cubic boron nitride, *Appl. Phys. Lett.*, **81**, 643, (2002).

Jiang C., Lin Z., Zhang J. & Zhao Y., First-principles prediction of mechanical properties of gamma-boron, *Appl. Phys. Lett.*, **94**, 191906, (2009).

Krell, A., Vickers hardness and microfracture of single and polycrystalline Al_2O_3, *Kristall und Technik*, **15**, 1467-1474, (1980).

Leger, J.M., *et al.*, Discovery of hardest known oxide, *Nature*, **383**, 401-402, (1996).

Li, X., Tao, Y. & Peng, F., Pressure and temperature induced phase transition in WB_4: A first principles study, *J. Alloys Compd.*, **687**, 579-585, (2016).

Li, Z., & Bradt, R.C., The single-crystal elastic constants of cubic (3C) SiC to 1000 °C, *J. Mater. Sci*., **22**, 2557-2559, (1987).

Mukhanov, V.A., Kurakevych, O.O. & Solozhenko, V.L., Thermodynamic model of hardness: Particular case of boron rich solids, *J. Superhard Mater.*, **32**, 167-176, (2010).

Nishiyama, N., *et al.*, Fracture-induced amorphization of polycrystalline SiO_2 stishovite: A potential platform for toughening in ceramics, *Sci. Rep.*, **4**, 6558, (2014).

Rizzo, H.F., Simmons, W.C. & Bielstein, The existence and formation of the solid B_6O, *J. Electrochem. Soc.*, **109**, 1079-1082, (1962).

Soignard, E., Somayazulu, M., Dong, J., Sankey, O.F. & McMillan, F. High pressure-high temperature synthesis and elasticity of the cubic nitride spinel γ-Si_3N_4, *J. Phys.: Condens. Matter*. **13,** 557–563, (2001).

Solozhenko, V. L., Andrault, D., Fiquet, G., Mezouar, M. & Rubie, D. C. Synthesis of superhard cubic BC_2N. *Appl. Phys. Lett.* **78** 1385-1387, (2001).

Solozhenko, V.L., Kurakevych, O.O. & Oganov, A.R., On the hardness of a new boron phase, orthorhombic γB28, *J. Superhard Mater.*, **30**, 428-429, (2008).

Solozhenko V, Andrault D, Godec Y, Mezouar M, Ultimate metastable solubility of boron in diamond: Synthesis of superhard diamondlike BC_5, *Phys. Rev. Lett.*, **102**, 015506, (2009).

Sumiya H., Harano, K. & Ishida Y. Mechanical properties of nano-polycrystalline cBN synthesized by direct conversion sintering under HPHT, *Diam. Relat. Mater.*, **41**, 14-19, (2014).

Sung, C.M., & Sung, M., Carbon nitride and other speculative superhard materials, *Mater. Chem. Phys.*, **43**, 1-18, (1996).

Tanakaa, I., *et al.*, Hardness of cubic silicon nitride, *J. Mater. Res.*, **17**, 731-733, (2002).

Tang, M. *et al.* Superhard solid solutions of diamond and cubic boron nitride. *Scr. Mater.*, **66**, 781-784, (2012).

Yan, C. *et al.* Ultrahard diamond single crystals from chemical vapor deposition. *Phys. Status Solidi A*, **201**, 25-27, (2004).

Yoneda, A, Cooray, T., & Shatskiy, A., Single-crystal elasticity of stishovite: New experimental data obtained using high-frequency resonant ultrasound spectroscopy and a gingham check structure model, *Phys. Earth Planet Inter.*, **190-191**, 80-86, (2012).

Zarechnaya E.Y., *et al*., Superhard semiconducting optically transparent high pressure phase of boron, *Phys. Rev. Lett.* **102**,185501, (2009).

Zerr, A., Kempf, M., Schwarz, M, Kroke, E., Goken, M. and Riedel, R. Elastic moduli and hardness of cubic silicon nitride, *J. Am. Ceram. Soc*., **85**, 86-90, (2002).

Zhang J. *et al.*, Elasticity of cubic boron nitride under ambient conditions, *Appl. Phys. Lett*., **109**, 063521, (2011).

Zhang R.F., Lin, Z.J., Zhao, Y.S. & Veprek, S. Superhard materials with low elastic moduli: Three-dimensional covalent bonding as the origin of superhardness in B_6O, *Phys. Rev. Lett.* **83**, 092101, (2011).

Zhao W.J. & Wang Y.X. Mechanical properties of superhard diamondlike BC_5. *Solid State Commun.* **151**, 478-481 (2011).

Zhao, Y. *et al.* Superhard B-C-N materials synthesized in nanostructured bulks. *J. Mater. Res.* **17**, 3139-3145 (2002).
